# Manual of General Anatomy Applied to Physiology and Pathology

**Published:** 1844-07-01

**Authors:** 


### Manuel d'Anatomie Générale appliquée a la Physiologie et a la Pathologie. Par *L. Mandl*, &c. &c.

### Manual of General Anatomy applied to Physiology and Pathology. By *L. Mandl.* Paris. Baillière, 1843.

The importance of a knowledge of General Anatomy to the scientific medical, and surgical practitioner, is at the present day so universally admitted, that to say more on the subject seems entirely unnecessary. In the preceding number of this Journal, we took the opportunity, when reviewing a work on General Pathology, of entering somewhat fully into the great importance of a knowledge of that branch of medicine. We there endeavoured to point out the advantages which must result to the practitioner from those general views of disease, which an acquaintance with general principles must present to him, and the vast superiority which a practitioner, furnished with such general principles, must possess over the man who, confined within the narrow precincts of mere specialities, is obliged, when called on to treat a case of disease, to have recourse to his nosology, in order to find a name for it, before he can bring himself to prescribe the proper treatment. The arguments we then adduced to prove the great utility of a knowledge of general pathology, will hold equally strong, *mutatis mutandis*, in establishing the vast importance of General Anatomy, as a branch of medical study. Before we commence our analysis of the work now before us, we beg leave to say a few words on the subject of General Anatomy and its peculiar objects.

The study of Special or Descriptive Anatomy, which must precede that of General Anatomy, shows us that many of the circumstances which belong to any one organ, belong at the same time to several organs, and that thus several individual circumstances are common to many organs. Of the membranes, for instance, of which descriptive anatomy shows us the stomach is composed, some are common to it and to the intestines, to the bladder, to the uterus, &c. In like manner, in respect to any one of these membranes, a careful examination of its structure shows that, in many points, its organization is exactly similar to that of all other membranes—an extension of this view leads to further important and interesting results. All the arteries of the body, whatever their situation or size, are found to be composed essentially of the same substances, disposed in nearly the same order and form—the same may be said of the veins. All the absorbent vessels, all vessels of every kind—all the bones, muscles, and nerves—the whole external covering of the body, widely as their various structures differ from each other, present no material difference, as far as regards the organization of each particular class. Hence, various organs of the body are disposed into what are called common systems, which common systems are said to consist of common tissues. Thus, all the vessels are collected and arranged under one common class, called the vascular system, and so on. The material that enters into the composition of each of these systems, consists of a substance of a peculiar nature; but as this substance is more or less generally diffused over the whole body, entering as a constituent element into the various organs, it is termed a common tissue. Now, as the various tissues constituting the body differ from each other with respect to their vital properties, and as diseases are nothing but alterations of those vital properties, it is at once evident that an acquaintance with the tissues and their vital properties is indispensable to an accurate knowledge of the diseases with which such tissues may be affected. We know that in every organ composed of several tissues, one tissue may be impaired without any disorder of the others—for instance, a single tissue is frequently diseased in the eye, whilst the other tissues entering into its structure continue healthy. It is Bichat who has said, the more deeply we study diseases and their phenomena after death, the more forcible and impressive must be our conviction of the necessity of considering local diseases, not with respect to the viscera, the whole of whose structure they do not attack, but with respect to the different tissues which are separately affected. For the distinction of the animal body into separate kinds of texture, we are mainly indebted to the labours of Malpighi, De Graaf, Ruysch, and several others. All these persons, however, confined their attention to anatomy and physiology. It is to the Hunters and to Cullen we are principally indebted for the application of this distinction of tissues to pathology.

A still more successful effort to apply Histology to Pathology, was made by Dr. Carmichael Smith, in 1790. Some years after, Pinel, in his celebrated Nosographie Philosophique, made the distinction of the membranes and other animal tissues the foundation of his pathology. A little time after, Bichat, in his Traité des Membranes, and subsequntly in his Anatomie Generale, gave the most perfect arrangement, and most comprehensive view of the different organic textures of the human body, that had yet appeared. The use of the microscope has established a new era in the study of General Anatomy, or, as it may be now styled, ***Minute Anatomy***, or ***Histology***. Bichat and his predecessors contented themselves, for the most part, with analysing the several organs into the various tissues into which they were capable of being divided. By the use of the microscope, however, a great deal more has been attempted, viz. to unravel the very tissues themselves into their minutest structure. And no doubt much good has resulted to physiology, and much, it is hoped, will result both to physiology and pathology, from this minute investigation of the various structures and elements of the body. On this subject, however, we shall take the liberty of making a few remarks. The men who are now so zealously engaged in prosecuting microscopical observations, should remember, that the present is not the first time the microscope has been employed for anatomical purposes; they should bear in mind what has been the fate of the discoveries of the microscopical observers, with some few brilliant exceptions, and, holding this in remembrance, they would do well to consider what may have led to such a fate. All that the microscope can do is to discover particular facts ; now the discoveries of particular facts, which are the immediate result of observation, are in themselves uninteresting and valueless, unless they are combined so as to lead to some grand general result—the mere isolated facts of minute anatomy, or of botany, or of any other science—can never be available in advancing such science, unless the reasoning powers be exerted so as to give them a shape and form, and unless the judgment be employed so as to ascertain their imports and relations, and the place they are fitted to hold in that department of science to which they properly belong—it is one thing to be a microscopist, and another a physiologist—no microscope, however good, or however cleverly it may be used, can afford a dispensation from the labour of thinking.

Whatever credit is conveyed by the word "discovery," is well deserved by those who skilfully select and judiciously combine known truths, so as to elicit important and hitherto unthought of conclusions—theirs is the master-mind—whereas, men of very inferior powers may sometimes, by immediate observation, discover new facts empirically, and thus be of use in furnishing materials to the others, to whom they stand in the same relation, as the brick-maker and stone-quarrier to the architect. The microscope should be to physiology what the telescope is to astronomy. We know that by means of the telescope Galileo discovered the Satellites of Jupiter—but by the application of mind to this isolated fact a grand principle in physics was attained, viz. that the propagation of light is progressive and not instantaneous ; by a further application of mind these little bodies have been converted into very pretty little chronometers, and so employed for determining longitudes—one instance more of the application of mind to an instrumental discovery. Dr. Bradley by means of the telescope ascertained, that the fixed stars changed their places in the heavens in the course of the year, that is, that the fixed stars were not at all *fixed*—this was a puzzler and would have remained so, had he not set himself to work in order to account for it, After many fruitless efforts the happy idea occurred to him of combining the effects of the progressive motion of light, ascertained by Galileo, with the motion of the earth in its orbit, and he found to his great delight, not only that there was no anomaly in the thing, but that the phenomenon of this apparent motion in the stars afforded one of the most satisfactory arguments in proof of the Copernican system. Here we have conclusive evidence of the indispensable necessity of employing the powers of the mind, when we wish to make mere instrumental discoveries available to science. What Galileo and Bradley did in their departments of science, we say to the anatomical microscopist, "go thou and do likewise." Isolated facts, however numerous, can never form a science. It is only by the mental operation of comparing, combining and abstracting, that a science can be made out of this otherwise *rudis indigestaque moles;* when this is done, and only then, can it be truly said,

*Mens agitat molem et magno se corpore miscet.*

In the first six Chapters of his work Dr. Mandl lays down certain general principles regarding the physical and vital properties—Chemical Composition—the various structures and textures of the body, which, however useful and necessary in an elementary work on general anatomy, we shall pass over, as not likely to prove interesting to our readers. We shall therefore pass on to the 8th Chapter, wherein he considers the Development, Increase, and Regeneration of the Organic Systems.

### GENERAL LAWS OF DISTRIBUTION.

The manner in which the organic systems are distributed in the human body, is subject to certain generalities, usually called laws. These laws drawn up into form by anatomists, have not always been the expression of the truth: the imagination, preconceived ideas, and material errors have frequently laid down laws of a very fragile character. The exact knowledge of these generalities is much more difficult than is supposed at first; we shall cofine ourselves in this place to the enumeration of the best ascertained generalities regarding the distribution of the organic systems. The body is constructed symmetrically. The most perfect is that of the two sides of the body or the *lateral symmetry.* But this analogy may be demonstrated also either between the different portions of an organic system, or between the different regions of the entire body, as well between the superior and inferior part of the body, which constitutes *successive symmetry,* as between the anterior and posterior surface, or the *perpendicular symmetry.* It may be remarked, in general, that this resemblance is never absolute; one side usually has more or less advantage over the other. The systems presenting most symmetry are the nervous, osseous, fibrous, serous, muscular and glandular systems; there is less of it in the vascular, and least in the serous system. The organs of animal life are arranged more symmetrically and more uniformly than those of organic life. Bichat stated positively that the organs of animal life are symmetrical, whilst those of organic life are not so; Meckel, however, proved the incorrectness of this opinion. The different similar and elementary parts composing an organic system are placed most frequently in a spiral line. We see this law exemplified in the vertebral column, which describes several curves. The division of the systems adopted by the author is founded on their distribution. First we have systems diffused over the entire body, and forming an uninterrupted aggregate, and others which appear only in certain parts; hence the division of the systems into two classes. Three systems compose the first class. The systems of the second class are characterised by the presence in greater or less abundance or by the absence of the systems of the first class, and especially of nerves distinctly visible and of capillary vessels. The following is the classification adopted by the author:—

*Classification of the Organic Systems.*

A. General Systems, diffused uninterruptedly over the entire body.
- 1. Cellular System.
- 2. Nervous System.
- 3. Vascular System.

B. Particular Systems existing only in certain parts of the body.
- *a.* Destitute of nerves and of capillary vessels.
  - 4. The System of the Cutaneous Appendices.
- *b.* Destitute of distinct nerves and of large capillary vessels, visible to the naked eye.
  - 5. Adipose System.
  - 6. Serous System.
  - 7. Fibrous System.
  - 8. Cartilaginous System.
  - 9. Bony System.
- *c.* Provided with distinct nerves and large network of capillary vessels.
  - 10. Muscular System.
  - 11. Glandular System.
  - 12. Cutaneous System.

FORMATION OF THE ORGANIC SYSTEMS.

The manner in which the tissues and systems of the human body are formed may be studied at two very different epochs: during fœtal life and after birth. In the fœtus, *the development of the organic system* may be examined as a whole, or *the development of the elementary parts* constituting it, may become the subject of examination. In the body considered after birth, we may investigate the manner in which *the increase, decrease* and *regeneration* take place. By *development of the organic system* is meant the formation of the system in the fœtus. Every development in which isolated parts are produced, supposes an organising base which contains the germ; this base is called *blastema.* Every tissue has its particular blastema, the development of which, in the superior animals, depends on the development of the entire body.

All the systems do not appear at the same epoch. The vessels and nerves are the first parts delineated in the blastema: the intestinal canal commences its formation nearly at the same time. The organs of the senses and those of generation present themselves at a more advanced period. Then the muscles, bones, and teeth appear, and last of all the cutaneous system with the tegumentary appendices. At first, the entire body is whitish and even transparent; the colour of the organs is developed only gradually. In the first period of their existence the systems are characterised by very great softness; it is only by degrees they assume the normal consistence. The traces of the primitive formation continue in some parts during the entire course of life; whilst, in others, no trace whatever can be discovered. According to Serres every organ is primarily formed of two lateral halves tending to unite on the median line. This is the law of centripetal formation. The liver commands in some degree all the evolutions observed in the viscera of the abdomen and chest. Placed in the middle of these two cavities, it is by it and around it that everything is arranged and disposed; its position assigns their position to the other viscera. The opinion, that the degrees of development which the human body passes through from its first origin to the moment of its perfect maturity, correspond to constant and permanent formations in the animal series, has now been proved to be false.

### CELL THEORY.

The author next proceeds to examine into the way in which the elements of the different systems take their origin, grow, and become transformed, so as to constitute the different tissues of the human body. This branch of anatomical study is mainly indebted for its present state of comparative perfection to the microscope. The origin of the cells in plants—the manner in which these become transformed, so as to compose the different elementary parts, &c. become subjects of great attention—this circumstance tended considerably to facilitate the study of the development of the tissues of animals. Raspail was probably the first, who insisted on the identity of development in the vegetable and animal tissues, by establishing his spiro-vesicular theory. Everywhere, he says, a mother-vesicle produces other internal vesicles, attached by the hilum and capable, in their turn, of becoming mother-vesicles. The vesicles become transformed, as in plants, into fibres, spines, &c.; the number, the position, &c. of the internal spirals, &c. produces the different organs. Dutrochet also insisted on the analogy existing between the development of the two organic kingdoms. In his first researches into histology, Valentin observed that the primordial mass of all the tissues was constituted by peculiar granules found in a transparent gelatine; he pointed out the difference of these granules in the serous and mucous structures at the period when these are being separated in the blastoderm. In the vascular system he found large globules or cells, which, from their form and aggregation, he compared to the cellular tissue of plants. He also called attention to the analogy in form between cartilage in a state of ossification, and the cellular tissue of plants. Valentin described the round cells of the globules and the intercellular substance placed between them in the chorda dorsalis of young embryos. Simultaneously with the zoologists, the botanists also directed their researches to the development of plants. J. Brown, in 1831, made mention of the nucleus of the cells of plants; Schleyden followed up these researches, and established a theory of which we here subjoin a resumé. In the gum of plants there appear at first some corpuscles, either mucous, or others somewhat larger, with more distinct contours. Around these corpuscles there are formed granulations which, with the enclosed corpuscles, Schleyden has called *cytoblasts.* The cytoblasts enlarge; when they have attained their full size, there arises at one of their surfaces a very thin transparent vesicle: this is the young cell, which soon becomes more consistent and larger, so that the cytoblast appears in the form of a nucleus; this nucleus is subsequently absorbed. Immediately after the publication of Schleyden's works, Schwann applied himself to the study of the animal tissues: he completed the analogies resulting from the observations previously cited, by stating that the primordial gelatinous mass of the tissues consists in cells, and that the bodies found in them are nuclei. One sole principle of development, says Schwann, *scil.* the cellular formation, is the basis of all the organic tissues, however different they may be: that is to say, Nature never collects the molecules immediately into a fibre, a tube, &c. but she always at first forms a round cell, and then transforms it, where it is necessary, into different elementary formations, such as we see in the adult state. The formation of the elementary cells is accomplished in all the tissues, with respect to the principal points, according to the same laws. The ulterior formation and the transformation of the cells are different in the different tissues.

The fundamental phenomenon of the formation of the cell is thus described: at first there exists an amorphous substance, the *cytoblastema,* which is found in cells already existing or outside them. Generally, small spherical corpuscles, *nucleoli,* appear first in the cytoblastema: a substance of greater or less thickness and very finely granulated, is then precipitated; and including one or two of these corpuscles, it forms small round or oval masses, spherical or flattened; these are the nuclei of the cells. These nuclei are small opaque, granulated bodies, seldom smooth or transparent, being from $\frac{1}{100}$ to $\frac{1}{200}$ of a millimetre in diameter; they are solid or hollow: in the latter case they are constituted of a thin, smooth, amorphous membrane, and their contents are limpid or finely granulated. Around these nuclei the cells form in the following manner: there is precipitated at the external surface of the nucleus a layer of a substance consisting of very fine granules, and different from the cytoblastema which surrounds it. This layer is not at first very distinct; but, by degrees, it undergoes changes by intus-susception, it becomes more distinct and more solid, and its external surface is changed into the membrane of the cell. This membrane at first surrounds the nucleus; but by little and little it becomes enlarged, and in the different cells there is developed a substance of a different chemical composition: the nucleus remains attached somewhere to the inner side of the membrane; but, at a later period, it generally disappears. The cytoblastema is deposited either outside, or within cells: the new primitive cells are formed, consequently, either in cells which already exist, or outside in the intercellular substance. With respect to the place where the new cells are formed in a tissue, the following is the law: they always appear at the point where the nutritive liquid penetrates in the first place into the tissue. Hence the formation of new cells, in the unorganized tissues, can only take place at the points of contact with the organized substance; whilst, in the tissues completely organized, where the blood is distributed through the entire tissue, it may take place through their entire substance. Schwann distinguishes in the cells two sorts of phenomena: the plastic and metabolic phenomena. The former are manifested by the formation of the cells in the cytoblastema; the latter, called forth by the metabolic forces, are the chemical transformations of the contents, produced by the solid parts of the cell, that is, by the nucleus and membrane. The way in which the cells become transformed into the elementary parts of the different tissues varies very much. The most important differences are the following:—1st, elongation of the cell into fibres, resulting probably from the circumstance that several points of cellular wall grow more strongly than the others; 2nd, the division of an elongated cell into several isolated fibrils; 3rd, the fusion of several simple or primary cells into a secondary cell. Schwann divides the tissues, according to the development of their elements, into five classes:—I. Individuality of the cells. 1st, isolated cells. *a.* In the liquids: globules of the blood, mucus, pus, and lymph. *b.* In the tissues: ganglionic corpuscles. 2nd, agglomerated cells: pigment, fat, horny tissue, crystalline. II. Fusion of the cells. 3rd, re-union of the membranes which belong to the cells; individuality of the cavities: cartilaginous, osseous, dental tissue. 4th. The cells become elongated and form fasciculi of cylindrical fibres: cellular, fibrous, and elastic tissue. 5th. Complete fusion of the membranes and cavities which constitute the cells: muscles, nerves, capillary vessels. Valentin states moreover that, similar to that which takes place in the vegetable kingdom, there is seen in the animal kingdom a granulated nucleus, containing one or more corpuscles, surrounded by an envelope (cell) more or less independent; this latter consists in a separate wall, and distinct contents. All the tissues, however heterogeneous they may be in their state of perfect development, result from this fundamental formation. The different ways in which this development takes place, are referred to nine fundamental types. Their enumeration we shall here omit. So important and extensive is this cell-formation, that according to the observations of J. Müller, Henle, and Valentin, it presides also over all new pathological formations. It is proved that all their fibres arise from cellular fibres. The general primary form then of all the tissues is the cell, a product of the transformation of the nucleus, whose existence is preceded by that of the *nucleolus*. The young cells, that is, the cells in the early periods of their existence, are soluble in acetic acid, whilst the nuclei remain insoluble. The author here subjoins the results of Henle's observations regarding the metamorphoses of the nuclei, and concludes this part of his subject with some reflections on the cell-theory. This theory he shows to be founded by analogy on Schleyden's observations regarding the structure and development of plants, observations too far from being adopted by all botanists. However the truth of Schleyden's remarks would prove nothing in favour of this assumed identity in the structure of animals and vegetables. The author thinks that the advocates of the cell-theory have sometimes allowed their imagination to be carried away by certain appearances; the observations have been interpreted in a forced and arbitrary manner, whilst other facts have been passed over in silence which were found to be opposed to the ideas already taken up. There is not a granule, which, under the eyes of some observers, has not been transformed into a *nucleus* or *nucleolus;* at other times, globules of fat have been taken for cells, with the nucleus absorbed. He next examines the principles on which the cell-theory is based. To the question, whether the microscopical elements of the animal tissues are cells, he answers in the negative. The term *cell* should be applied only to elementary parts which admit a clear distinction between parietes and contents. Now this occurs very rarely in the animal tissues. In most instances we meet lamellæ or solid corpuscles penetrated with an organic liquid. This liquid is not collected in an empty space within the corpuscle, since no such space exists, but it penetrates equally all its organic woof. So that the name of cell is inapplicable to most of the elementary parts.

The most general process by which the elementary parts become developed are, according to the author, as follows: The liquor sanguinis (le liquide sanguin) transuding through the parietes of the vessels, occasions the coagulation of the fibrine dissolved in this plasma. The fibrin is coagulated in the form of small corpuscles, to be hereafter described under the name of *fibrinous globules*. These corpuscles become the centre of a new formation; a solid matter is thrown down gradually around these corpuscles, which in this way become surrounded by a solid substance different from that of the fibrinous globule. This matter proceeds from the coagulation of the blastema or primary organizing matter. Attraction takes place chiefly in two directions, in length and breadth; the thickness is inconsiderable. In this manner the primary corpuscle, the fibrinous globule, is transformed into a new element, two dimensions of which have greatly the advantage over the third. This form is so much the more remarkable, as the same law is also observed in the dimensions of the entire body. The new elements are either lamellæ or corpuscles; they present the primitive corpuscle lodged in their substance, sometimes on their surface. From its position we shall call the latter the nucleus of the lamella or corpuscle. This nucleus may still enlarge after the formation of the lamella. It was stated that the primary corpuscle, the nucleus, in the theory of cells, arises from the coagulation of the transuded liquor sanguinis. But we may admit that a similar coagulation may also take place in the midst of the blastema: the corpuscle, produced from this coagulation, would in its turn become a centre of new attraction. The attraction around the primitive corpuscle cannot surpass certain limits, and the matter thrown down becomes more and more consolidated, and shortly becomes quite distinct from the blastema in which it is plunged. In some cases the external surface is better consolidated, than the rest of the corpuscle, and in this way occasions the production of an external membrane; this membrane, however, is only of subsequent formation, and frequently cannot be clearly distinguished from the entire element. An example of this formation presents itself on studying the texture of the grey substance of the nervous system. It will be seen that the grey corpuscles exist at first, that the grey matter is precipitated around these corpuscles, that it gradually becomes solidified, and that in some cases there is even formed an external membrane. The process of the formation of the ovum appears analogous. At the same time, or before the matter is precipitated around the corpuscle, changes occur in its interior which give rise to that which the abettors of the cell-theory have called the *nucleolus*. These elements appear to us then to be the result of a secondary formation. In some cases the matter precipitated around the primitive corpuscle, forms concentric layers; at other times, this corpuscle remains excentric, that is, the matter precipitated rises over the corpuscle, like the glass of a watch.

The author next makes some remarks on the increase and decrease of the organic parts, on the regeneration of tissues which may have been destroyed, and on the differences subsisting between man and other animals, between one man and another, and also the differences observable in the same man at different periods of life, and then proceeds to consider the results of what has been said with respect to physiology and pathology.

The facts acquired to science by the researches of general anatomy find their application in the examination of the physiological and pathological phenomena of the human body. The explanation of these phenomena rests partly on the facts made known by general anatomy. The study of the physical, vital, chemical and anatomical characters will frequently afford an opportunity of speaking of the results acquired to physiology and pathology. The most important result of these researches is no doubt the knowledge of the organic systems, a knowledge which explains why parts very remote from each other are simultaneously affected. The attention of medical men to these circumstances was first called by Pinel. It was these ideas that formed no doubt the first basis of modern medicine, and of the researches of Bichat.

### ANOMALIES.

The congenital deviations from the normal type sometimes seen in the body, either in its external forms, or in the distribution of the organic systems, present very great differences. When the anomaly exercises no influence on the performance of the functions of the organ which is its seat, and that it does not at all modify the external form of the body, it is called *anatomical* variety. Such, for instance, is the presence of a supernumerary muscle—the existence of only one kidney—transposition of the viscera, &c. When the congenital deviation opposes the free discharge of the functions, so as to render the existence of the individual impossible, it constitutes a *monstrosity* properly so called. The ætiology of anomalies is derived from our knowledge of the development of the organic systems. The opinions which have been advanced in accounting for monstrosities may be referred to three principal hypotheses, which mutually support each other: these are the theory of arrest of development, to which is closely allied that of centripetal formation, and the opinion which attributes a creative power to the arterial system. According to the best anatomists and physiologists most anomalies depend on arrest of development: monsters, in their conformation, represent, by one or more of their organs, the transitory state of the embryo in its different periods of growth. M. Serres it was who laid down the law of centripetal formation. On this supposition every organ is primarily formed of two lateral halves, tending to unite on the median line. G. Saint-Hilaire applied this law to explain monstrosities. If the development is arrested, the union does not take place: hence we have median scissures. The development, on the contrary, being in excess, the organs then tend to unite on the median line: hence the fusion of organs and of cavities. Of the causes which produce abnormal development, the author is not disposed to treat. He merely states the most general conditions under which anomalies and monstrosities occur: for, in the midst of this apparent disorder, order still prevails; this cannot be understood but by a knowledge of the fundamental type. This may be called pathological general anatomy.

The author here lays down the general laws observed in the development of the various structural anomalies and monstrosities. Thus, 1. When two subjects become fused together, different tissues are never connected together: it is only similar organs are confounded when a monstrous juxta-position takes place. 2. In every monstrous individual there exists, between the different anomalies constituting it, a sort of organic balancing, by means of which an anomaly by defect draws on an anomaly by excess of development; as if there existed a certain amount of productive force, which, if it did not act on one side, must necessarily act on the other. 3. Anomalies are so much the more frequent, in proportion as they are less serious, and in the inverse ratio of the importance of the organ affected by them: thus, varieties are more frequent than monstrosities. In the distribution of the vascular system it has been remarked that varieties are more usual at the termination of the branches than at their origin. The anomalies most rarely met in the vascular system are those which occasion a mixture of the pulmonary blood with the blood of the body, and thus seriously affect the health. 4. Most of the defects of conformation occur on the left side; it seldom happens that the vertebral artery of the right side arises directly from the aorta, whilst this is of frequent occurrence on the left. There are, however, exceptions to this law. 5. Certain systems are more subject than others to the defects of conformation. The greatest portion of the nervous system, the osseous system, and the muscular system, present anomalies much more rarely than others, and more especially than the vascular system. 6. The more an organ is repeated in the body, the more obnoxious is it to anomalies, and the less important is the anomaly. It is observed that the anomaly by excess is more frequent in the upper half of the body than in the lower. 7. There is some analogy between the anomalies of one and the same organ; when, for instance, there are two tongues, they are superimposed, one upon the other, and not placed laterally. The perforation of the septum of the heart, the divisions of the urethra, &c., always occur in some particular point. 8. Every organ presents certain deviations rather than others. The kidneys, for instance, have a particular tendency to unite on the median line. 9. The same individual may present several anomalies at one and the same time. Sometimes they occupy organs belonging to the same apparatus; sometimes they have their seat in different regions of the body. Thus the heart is always wanting in acephalous fœtuses; the simultaneous disappearance of the brain and supra-renal capsules may be said to be constant, &c. 10. Defects of conformation are confined within certain limits: to whatever degree the form of an organ or of the entire organism may deviate from the normal standard, it never becomes altogether incapable of being recognized: the heart has never been seen on the back, the lungs in the abdomen, &c. 11. However great the monstrosity may be, it will never cause the fœtus to descend in the zoological scale. The erroneous opinions of some writers on this subject have had their origin in a supposed law, viz. that man passes through degrees of formation corresponding to constant formations in the animal series. 12. Defects of conformation are more frequent in the female; still, various defects of conformation of the heart and bladder occur more frequently in the male than in the female. 13. Certain anomalies seem to be hereditary: members of the same family having six fingers may be adduced as an instance of this.

The author next proceeds to an enumeration of the various morbid productions occurring in the various organs and parts of the body. He here adopts Müller's classification, with which our readers are already sufficiently acquainted.

In the *Second Section*, CHAP. I., he treats of the CELLULAR SYSTEM, which he defines to be a whitish or greyish mass, extremely soft, possessing but little resistance, moist, very elastic, diffused throughout the entire body, forming an uninterrupted whole, consisting of lamellæ and fibrils placed in juxta-position, the interstices of which are filled with serosity. With respect to its *Physical Properties*, it is very extensible; when very much distended, it at first becomes perceptibly attenuated, and at length gives way. This property of extensibility it owes to the presence of serosity. As soon as extension of the tissue ceases, retractility is produced. This is a distinct property from contractility, which is organic, and manifests itself after the employment of stimulants. The retractility in question is but a physical property, and the effect of elasticity. The cellular tissue is in general very soft; it is always moistened with serosity —it is colourless, when it occurs in thin layers; when it is thicker, it is whitish; it is semi-transparent, when distended. When dried by heat, it loses these physical properties, and acquires new ones.

*Vital Properties.*—Sensibility is not the attribute of the cellular tissue in the ordinary state. It may be cut, and drawn in different directions, without the individual experimented on evincing any sign of pain. The cellular tissue is endowed with organic contractility, that is, the property of shortening during life under the influence of certain irritating agents. This contractility is most perceptible in the tunic of the dartos appertaining to the scrotum; which has induced some anatomists to constitute this a separate and particular tissue. All the absorption which takes place in the cellular tissue must be attributed to the blood-vessels existing in it. We must take care to distinguish imbibition from absorption; the former is a physical phenomenon common to all the tissues; whilst absorption is an organic property, with which certain vessels only are endowed.

The *Chemical Properties* of cellular tissue are next considered at full length. Then the *Structure;* here the opinions of Bichat, Bordeu, Meckel, and others, with respect to the nature and structural peculiarities of this tissue, are freely canvassed. The author next notices the texture, distribution, and mode of development of the cellular tissue, which, as containing nothing new, we shall pass over and proceed to notice a few of the pathological relations of this system. A knowledge of the physical properties of the cellular tissue accounts for several pathological phenomena. The most remarkable of these properties is its permeability. This property will at once explain the following facts :—1. Migrations of solid bodies which readily traverse the cellular system. Pins, introduced into the stomach, or under the skin, are found to reach the fingers, toes, or other parts of the body. 2. The facility with which emphysema manifests itself after wounds of the lung, or after rupture of one of the cartilaginous rings of the trachea. Air penetrates very rapidly throughout all the cellular tissue, not only beneath the skin, but also in the interstices of the muscles, and in the substance of all the viscera. When a large aperture is made to favour its escape, it passes out only in very small quantity, owing to the filamentous structure of the cellular tissue retaining the air in its interstices. 3. The readiness with which purulent collections are removed to a distance. The matter of the abscesses developed in the upper part of the body, escapes even to the feet, through the cellular tissue which fills the interstices of the organs. 4. In general anasarca it not rarely happens that all the serosity escapes by one single aperture; in œdema we may drive the serum from one part to another; it accumulates more especially in the depending parts. Thus, the feet are observed to become infiltrated first, and it is only at a subsequent period the upper extremities become so. All these phenomena suppose, not only the permeability of the tissue, but also the great elasticity of its elements. This elasticity, however, is altered by the effect of inflammation, and of other morbid states which render the tissue fragile. Thus, the cellular tissue of the small intestine becomes so fragile in cases of chronic enteritis, that on the dead body of an individual who has died of this affection, one may draw off in one piece the whole mucous membrane of the intestine. The cellular tissue which surrounds the womb that has become cancerous, being engorged and tumefied, the least effort is sufficient to tear it.

Bichat was deceived in attributing to the cellular tissue an isolated power in diseases of different organs. This isolation of diseases of the organs depends chiefly on the structure proper to each tissue, and on the differences of the vital properties with which it is endowed. On the other hand, in acute diseases, which have their seat in a particular organ, in the lungs, stomach, intestines, &c., the cellular tissue is often affected by juxta-position, and by reason of continuity with the organ affected. It becomes the seat of inflammation, of collections of pus, &c. Inflammation and the production of pus have not their seat in the anatomical elements exactly of the cellular tissue: inflammation is solely an affection of capillary vessels: but the great softness and extreme permeability of the cellular system allows the pus to accumulate more readily there than elsewhere. We are not to suppose that the cellular tissue is transformed into pus by inflammation; this morbid secretion only displaces this tissue, which is gradually destroyed.

The isolation of the organs in disease, is to be attributed, in general, not only to the different vital properties, but also to the difference of the blood-vessels, that is, to the existence in these organs of vessels arising from different trunks: thus, we find the nerves entirely intact, though all around them may be destroyed by suppuration. The knowledge of the degree of cohesion, different in different regions of the body, explains several phenomena in disease. The density, for instance, of the cellular tissue on the nose, especially inferiorly, and on the alæ, produces remarkable phenomena in erysipelas of this region, and acute pains, occasioned by the difficulty with which the skin of the nose becomes tumefied. Surgical operations, ligatures of vessels, are more difficult in the midst of a dense tissue; thus, they are more difficult in the subcutaneous tissue of the nucha, than in that on a level with the larynx. Serous infiltrations, and emphysema take place more readily in the general tissue, and in that which is very lax, than in cellular membranes, or in dense portions of the cellular tissue. Hence, the rarity of serous infiltrations in the palms of the hands, or soles of the feet. We rarely see serous infiltrations or emphysema in the dense cellular tissue beneath mucous membranes. Serous infiltration of the cellular tissue of the large intestine is rarely observed in chronic diarrhœa.

The author next considers the General Anatomy of the *Nervous System.* This system he divides into two parts; one he calls the *central part,* or the central nervous system; it is inclosed in the cavities of the cranium or spine. The other part of the nervous system, *the peripheric part,* constitutes the nerves. Of these, there are two classes; the one arises from the spinal marrow by two roots; these are the *cerebro-spinal* or *white nerves.* These, including the central part of the nervous system, are designated *the cerebro-spinal system,* or the nervous system of animal life. The others form a chain of soft nerves, and grayish-red ganglions; this is *the ganglionic system,* or the nervous system of organic life. Of the *physical properties,* the colour is the most important in this system. The white and gray colours are those which predominate, and serve to distinguish the different parts of the central portions of the nervous system. The gray and white substances of the nervous centre differ essentially from each other, in their structure and their texture, in some of their physical properties, and probably, also, in their functions. That the nervous substance possesses extensibility, to a certain degree, is proved in the case of dropsy of the ventricles. The other physical properties we shall not dwell on, but proceed to consider the vital properties. The essential vital property of the cerebro-spinal nervous system, and which solely appertains to it, is sensibility. The peripheric portion, which comprises the nerves properly so called, is sensible to a high degree. The seat of this property is, beyond all doubt, the medullary substance, the neurilema being composed merely of cellular tissue, and its removal not being sufficient to cause pain. These nerves are the organs of sensation and motion. With respect to the sensibility of the cerebral substance, which is considered as the seat of sensation, motion, and of the functions of the mind, opinions are divided. From experiments made by several physiologists, the author concludes that the brain, in the ordinary state, has no consciousness of the irritations addressed directly to its substance, and it appears, that the impressions, in order to be appreciated by this organ, require to be transmitted to it through the medium of the nerves. All are agreed as to the sensibility of the medulla spinalis and medulla oblongata.

We shall now pass on to the Chapter on the BLOOD, in which we find the author has collected all the more modern discoveries with respect to this important fluid. We shall first consider *the physical and vital properties of the blood.* The arterial blood is of a red vermilion colour, whilst venous blood is a deep dark colour. The *heat* of the blood, examined in the vessels, is equal to that of the body; it is therefore 38 C.; its caloric capacity, that is to say, the rapidity with which it cools, is related to its specific gravity. Almost all observers have found that the arterial blood is from $1\frac{1}{5}°$ to $1\frac{1}{2}$ C. warmer than venous blood. Its specific gravity varies between 1.050 and 1.059; the mean is 1.055; arterial is lighter than venous blood. The *quantity* of the blood has been determined by some at 10, by others at from 12 to 15 kilogrammes. Valentin says that it is to the weight of the entire body in the ratio of 1 to 4.25. The consistence of the blood depends on its specific gravity, heat and viscidity. When the blood is drawn from the vessels, its consistence changes; it becomes solid, that is, it coagulates: it is at first a gelatinous mass, which then contracts, and in this way forms the *clot* and the *serum* or *serosity*. Arterial blood coagulates more quickly than venous blood. With respect to the *cause of the coagulability* of the blood various opinions have been advanced. Some will have it that the blood is kept in the fluid form by its inherent *vitality*. Aneurysms, however, afford an instance of blood coagulating in the vessels during life. Others refer the coagulation to the *loss of motion and of heat*. This, however, has been disproved by repeated experiments, as also by pathological observation.

Almost all authors have thought that the external *air* may produce coagulation of the blood, a great many experiments go to prove that coagulation is accelerated by favouring the access of the air, and that it is retarded under the opposite circumstances. These experiments do not seem deserving of much confidence from the difficulty of completely excluding the contact of the air with the blood. The coagulation has been attributed chiefly to the oxygen, not to the azote, whilst the carbonic acid gas retards or prevents it: a proof of this has been sought in the blood of asphyxiated persons, &c. It is now ascertained that the blood contains several species of gas: venous blood more especially contains carbonic acid gas; an attempt has been made to favour the development of this gas by placing beside the blood some lime-water to see whether the coagulation would be accelerated, thus attributing to the development of the gas the cause of the coagulation. Home and Scudamore concurred in this view of the matter. Other physiologists imagine that the formation of the carbonic acid gas by oxygen should be considered as the real cause. The author considers the two last opinions the most probable. Hewson was one of the first who ascertained that the sulphates, muriates, nitrates, phosphates, acetates, borates, and the carbonates of soda and of potash, when mixed with the blood, are capable of preventing its coagulation. The carbonates are the most powerful; free alcaline solutions are long known to possess this preventive power. The author has seen similar effects produced by the presence of pus and albumen. In all these researches there are two circumstances which must not be forgotten; 1, that almost all substances mixed with the blood in very small quantity accelerate coagulation, even when in larger quantity they retard it. Soda and caustic potash form exceptions to this; the minutest quantities of these substances retard coagulation: 2, it must be remarked that large quantities of foreign substances may retard coagulation, by their preventing the particles of fibrine from approximating. The results of modern experiments on this subject may be thus summed up: 1, the alkalies, potash, soda and ammonia, entirely prevent coagulation; lime retards it; 2. Soluble alkaline salts (combinations of soda, potash, ammonia, magnesia, baryta and lime, with carbonic, acetic, nitric, phosphoric, tartaric, boric, sulphuric and cyanhydric acids, as also the chlorides) in very small quantity, favour coagulation. These substances, on the contrary, when in a concentrated solution, retard and even entirely prevent it. The most active salts are the carbonates; the least active, the combinations of chlorine and the sulphates; 0.007 of carbonate of soda retard coagulation for several hours, whilst the sulphates do not act in the proportion of 14 to 1000. The action of a salt is so much the more marked, in proportion as it reddens the blood more, whilst the combinations of chromine, chlorine, and iodine do not at all redden it, and do not prevent its coagulation. When to blood liquified in this way by a salt there is added a greater or less quantity of water, a new coagulation takes place (fibrine is precipitated). 3. The metallic salts decompose the blood, and sometimes produce a coagulation, sometimes prevent it. 4. Very dilute vegetable acids favour coagulation; when a little more concentrated, they prevent it; when very concentrated, they decompose the blood, like the mineral acids. 5. The other vegetable substances have not yet been sufficiently studied: some say, for instance, that narcotics prevent the blood from coagulating; others affirm the contrary. We are in the same doubt regarding the action of poisons; it is, however, generally believed that the latter, as well as thunder, a violent electric discharge, the instantaneous destruction of the nervous system, &c. prevent coagulation. 6. Very dilute solutions of gum Arabic, of sugar, albumen, milk, &c. appear to act merely in a mechanical way in preventing coagulation.

From the time the blood becomes coagulated, it goes on contracting until the clot floats in the serosity. It is difficult to determine the quantitative relations between the clot and the serum, as also to ascertain the time necessary for all the serosity to be expressed. The contraction is sometimes terminated at the end of six hours; sometimes not till a much longer period. The more watery the blood is, the less will it contract.

When the blood is prevented from coagulating, by suspending in it some portion of animal substance, such as a piece of intestine, there soon forms a deposit of red particles, over which a colourless fluid floats. This is distinct from the serosity of the blood. This is called the *plasma, liquor sanguinis, plastic lymph*; it is colourless, a little turbid and viscous.

The author next proceeds to consider the *Chemical Properties of the Blood.* The number of substances constituting the blood varies, according to different authors, between 20 and 29. It also contains gases in solution.

Having noticed the various ways by which fibrine and albumen are obtained from the blood, he then considers the differences between these two substances. Mulder was the first who proved that these two substances are modifications of *proteine.* According to the same authority, fibrine contains more oxygen than albumen does. Vogel affirms the contrary; the latter chemist has also found less azote than in albumen. Mulder states that phosphorus and sulphur enter into the elementary composition of fibrin and albumen; that the latter contains almost twice as much sulphur as the former, the quantity of phosphorus being the same in both. Fibrine coagulates spontaneously, whilst albumen requires an elevated temperature to make it do so.

He next details the re-action of chemical agents on fibrine and coagulated albumen. M. Lecanu has succeeded in separating the red colouring matter of the blood into two parts, the one colourless, the other coloured red. Berzelius calls the former *globuline*, the latter *hematine*. Globuline appears to be a substance intermediate between albumen and caseine—it contains phosphorus and sulphur. Hematine is insoluble in water and alcohol—all the iron of the blood seems to be combined with the hematine. It is not known under what form the iron exists in the blood, nor whether the blood owes its colour to this metal.

With respect to the colour of the blood and the changes it undergoes, whether by gases or different chemical substances, the coagulum floating in the serum has a bright red on the surface, whilst the bottom of the coagulum acquires a deeper colour. The oxygen reddens the blood very much; carbonic acid renders it black. Generally speaking, the blood acquires a deeper colour by alcalies, a brownish colour by acids, and greenish by chlorine. All the substances which combine with the oxygen of the blood, render it more blackish. The neutral salts, especially nitrate of ammonia and the hydrochlorates, give the blood a brighter red colour. The *yellow colouring matter* of the bile it is, which gives to the serum of the blood its greenish yellow colour. The other elements of the bile have not been found in the blood even during jaundice. The author now considers the leading differences between arterial blood and venous blood. Hering, Simon, and Nasse say that arterial blood contains more water. Lecanu and Letellier affirm the contrary. Lecanu also states that arterial blood incloses more blood-globules than venous blood. The quantity of fibrine appears to be more considerable in arterial blood; the fibrine of venous blood is softer, and deprived of colour with more difficulty; that of arterial blood is soluble, according to M. Denis, in nitrate of potash, but not that of venous blood. The quantity of albumen, of fatty and extractive matters, and of salts, is nearly the same in both kinds of blood. The author next enters most fully and satisfactorily into the microscopical examination of the blood. We shall present here a succinct analysis of the results of this examination.

*Red corpuscles, Blood-globules.*—The blood-globules of man are small round discs, the central part of which appears shadowed or transparent, according as it is approximated to or removed from the focus of the lenses. On observing some of these corpuscles placed on the field of the microscope, one soon becomes convinced that this central part is but the thinnest part of the globule. This globule is depressed on both sides, since, when placed on the field of the microscope, it presents the appearance of an elongated 8. The edge forms a thick ridge all around, more coloured than the central part; it occupies nearly one-fourth of the diameter of the blood-globule; but in the globule floating in the serum, it is not separated by any line from the central portion. This central depression is however not to be considered characteristic, nor essential to the form of the globule. The blood-globules of most of the mammalia have the same form as those of man; but their size varies very much in different families. The elephant has the largest globules amongst the mammalia, and the ruminantia the smallest; the family of the chameaux is the only one whose blood-globules are not round as in the other mammalia; on the contrary, they are elliptical, as in birds, reptiles and fishes. In the latter, *scil.* birds, reptiles and fishes, the blood-corpuscles appear at first as if united together; after a while, however, a nucleus is seen to appear on the centre, which becomes more prominent, the longer the globule remains on the porte-objet. When placed on the field of the microscope, the globule presents no central depression, but rather an elevation, which, however, is not an abrupt prominence; it continually diminishes towards the two extremities of the corpuscle. This prominent part of the elliptical globules is called *nucleus*. In the round globules there is only found at times a small central granule, which at other times does not exist, or is produced only by drying the globules. Hence the different opinions of authors regarding the presence or absence of a central nucleus in the globules of the mammalia. In the globules, then, we may distinguish the nucleus and the membrane. The membrane is the seat of the colouring matter, the hematosine of the globules, which is deposited not in granules, but continuously all over the corpuscle. When treated with water, the membrane is not dissolved; but it is deprived of colour, and it is only with difficulty or by the aid of colouring matters (tincture or vapour of iodine) that its presence can be ascertained. The coloured matter appears neither inclosed within the membrane, nor deposited on its surface, but combined with the substance itself. Neither is the latter to be considered an envelope including fluid contents; but the entire membrane appears to consist of a single substance, filling up the entire thickness of the corpuscle, coloured continuously, and floating in the liquor sanguinis. The corpuscles, not being hollow internally, cannot therefore inclose either water or gases. This last opinion, however, which is that of Schultz and Berres, is at once refuted by the appearance of the globule, the presence of gases being made manifest by the particular manner in which they refract light. This membrane cannot be proved to be denser externally than internally; so that there is no reason for calling it an *envelope*, except the better to distinguish it from the nucleus.

The *nucleus* is probably colourless; it is oblong in the elliptic globules, and seems composed of several small granules; in such of the round globules as have it, it is round, composed of a single small granule. It usually occupies the centre of the globule: its diameter is from one-fourth to one-fifth the length of the globule. The author seems inclined to think that the nucleus is formed chiefly by coagulation; because, at first there is scarcely a trace of nucleus, and it becomes the more apparent, the longer the globule has been out of the body. He admits, however, that at times there exists in some globules a slight trace of nucleus even at the moment when they are placed beneath the microscope, or even during the circulation. In the nucleus, there exist some granules described as *nucleoli* of the nucleus. These granules seem to arise from very minute drops of oil, inclosed within the nucleus. Müller, Simon, and Maitland think that the nucleus is formed of fibrine. The colouring matter is for the most part deposited in the blood-globules; but a portion is also found dissolved in the liquor sanguinis, which is always a little reddish. The round blood-globules have a great tendency to adhere together by their flat side, so as to form piles, like pieces of coin. They are also very elastic in all vertebrated animals, so that their longitudinal diameter acquires sometimes double its length. The changes which take place in the globules by drying, by their decomposition in the serum of the blood, or by the action of foreign substances, are very remarkable, and merit all the attention of the physician, as a knowledge of these points may be turned to good account in pathology.

The *decomposition* of the blood-globules takes place very rapidly, especially in Summer; it is probably produced by the alteration of the serum. The globules become serrated at their edges; folds are formed, which are continued for some distance towards the centre, and which have been taken by some for internal compartments. By degrees these folds divide the membrane of the globule more deeply; the edge then seems to consist of small granules (from 8 to 10 in the human subject). *Desiccation* also produces indentations on the edges; whilst the central part of the round globules is cleft by desiccation, in the elliptic globules the nucleus becomes more apparent, and at the same time blackish. *Water* transforms the blood-corpuscles of man and of the mammalia into real round globules, the globule is at the same time deprived of colour, becomes grayish and very transparent; some chemical agents may serve to render it perceptible again. When, for instance, the coagulum of the blood of a mammiferous animal is washed until the form of the globules can no longer be distinguished, we have only to add the acetate of lead to make the membranes of the globules re-appear. *Hydrochlorate of Soda* produces indentations on the corpuscles and transforms them into round globules smaller than those produced by the action of water. *Æther* renders the corpuscles smaller, paler, and at length dissolves them almost entirely. *Bile* and *Urea* dissolve the corpuscles according to *Hünefeld*. *Dilute albumen* changes their form more than pure water. It appears that *carbonic acid gas* renders the corpuscles more opaque, whilst they become more transparent in oxygen.

*White Globules.*—Müller was the first to notice these in the blood of frogs, and the author in the blood of the mammalia. They are small colourless, finely granulated corpuscles, insoluble in water, and strong refractors of light. There are two species of them: some are round, and include two or three granules, which become more apparent, when treated with acetic acid; these are true *lymphatic globules*, arising at least in part from lymph mixed with blood. The others also are generally round though sometimes oblong; at other times irregular, with edges slightly serrated, having a finely granulated surface. These globules are the product of the coagulation of the fibrine, whence called *fibrinous globules*. The author thinks it probable that the fibrinous globules are transformed, during the circulation, into globules of the first species, so that the latter do not seem all to come from the lymph. We have found their quantity increased in fever patients who had not eaten for several days, in phthisical patients, &c. There are several forms of transition between these two principal forms.

*Molecules of Coagulated Albumen and Drops of Fat.*—Albumen in the state of solution is a limpid liquid, presenting no species of granules; but when it is coagulated, there is presented a multitude of molecules, either isolated, or combined in great quantity. Some of these molecules exist also in the blood, and seem to arise from the albumen coagulated by the salts contained in the serum. Some few drops of *fat* exist at times in the serum ; crystals form there only by desiccation.

*Liquor Sanguinis, Plasma.*—Such is the name given to the liquid in which the corpuscles are suspended ; it is colourless, though sometimes it presents a slightly reddish tint. It may be asked what relation exists between the globules, the coagulum, the liquor sanguinis, and the serum. Microscopical observation had already proved that the coagulum contains blood-globules entire.

Müller succeeded in separating the globules and the liquid which holds them in suspension. The coagulum is formed notwithstanding this separation ; hence it is evident that the blood-globules go for nothing in its formation. The difficulty was to find a filter fine enough to retain blood-globules. Human blood cannot be filtered. But the blood-globules of the frog remain on the filtering paper, whilst the liquor sanguinis passes through, and soon coagulates into a white, limpid coagulum, entirely consisting of fibrine. This then is dissolved during the circulation in the liquid which holds the globules in suspension, and which may be called the liquor sanguinis to distinguish it from the serum (the *plasma* of Schultz) to distinguish it from the serum which holds no fibrine in solution.

The author now passes on to the consideration of the distribution of the blood, that is, its circulation; then to that of its development, to the differences occurring in it, and depending on age, sex, and a variety of other circumstances; then to the development of its several elements. All that has been said of the way in which the blood-globules are developed in the embryo is so hypothetical, that we do not deem it necessary to enter into detail on that subject. The study of the development of the blood-globules in the adult has yielded to Nasse, Wagner, Henle, &c., chiefly this result, *scil.* that the red corpuscles arise chiefly from a transformation of the white globules. The elliptical blood-corpuscles undergo the following changes :—1, they are at first white, (lymphatic,) round globules ; 2, these white globules are surrounded by a very thin round envelope ; 3, they become elliptical pale corpuscles, and but little changed by water; 4, lastly, the nucleus becomes elliptical, and the globule coloured. The round blood-corpuscles present the following degrees of development :—1. They are white globules insoluble in water. 2. A nucleus is formed in them. 3. They become lenticular corpuscles with a nucleus which is divided into others that are smaller. 4. These become transformed into flattened corpuscles with a divided nucleus, and into others with a lateral impression. 5. Hence arise flattened, very pale corpuscles, not much changed in water, which at length become—6. Perfect blood-corpuscles.

The author avows that his researches on the blood-corpuscles are not yet brought to a close. He next comes to the regeneration of the blood (sanguification). The intestinal tube is, during extra-uterine life, the principal source of sanguification. There is formed in that organ, by the assimilation of the digested aliments, a liquid which serves for the regeneration of the blood. This liquid bears but slight resemblance as yet to the blood during its sojourn in the chyliferous vessels; but, according as it is advanced slowly through the vessels, the mesenteric glands, and the thoracic duct, its resemblance to the blood becomes more and more marked, until that passing into the venous system, it reaches the heart, intimately mixed with the venous blood. A portion of this liquid, however, is absorbed by the vessels of the intestinal tube, and mixed with the blood of the vena portæ.

The lymph may also be considered as another source of the blood ; but the fluid itself takes its origin from the blood which has transuded through the vascular parietes, and has not served for nutrition. With respect to sanguification, two species of lymph may be distinguished ; the one, the less perfect, returns from all the parts of the body, and has to pass through the lymphatic glands before it can reach the blood ; the other is the chief product of what are called the sanguineous glands. The lymph passes immediately into the blood, or only after having been mixed with the chyle. This last circumstance takes place for the lymph of the lower extremities, of the uro-genital apparatus, of the spleen, and of the supra-renal glands.

The lymph and chyle (white blood of some writers), mixed with the blood, reach the heart, where certain physical and chemical changes are probably produced. Then passing into the lungs, it becomes fitted for nutrition by the rejection of carbonic acid gas, and by the access of oxygen. The action of the respiration appears to be exercised chiefly on the liquids mixed with the blood. Hence the principal sources which furnish new substances to the blood, are the intestinal tube and the lungs. The blood, passing through the different secreting organs, furnishes the elements of secretion. Some of these are entirely lost to the blood ; such are the secretions of the kidneys (the urine), of the lungs (gas, water), and also of the skin. Other secretions, on the contrary, partly return to the blood ; as the secretions of all those glands whose excretory ducts are placed on the surface of the digestive passages, as the saliva, pancreatic and gastric juices, &c.

After having in this way given a general view of sanguification, he then proceeds to examine separately in what way the different elements of the blood are formed. Most of the solid elements are combinations of *proteine.* The question is to know whether all the proteine comes as such, already formed in the digestive canal, or whether it is not, at least in part, the product of this transformation of the substances digested. To the present time persons believe in these transformations, for instance, of albumen into fibrine, &c. ; in other words, it is supposed that, by the vital forces, the less azotised substances become more rich in azote, according as they are transformed into blood. But new researches are tending to prove that all the materials of the blood exist already formed in the aliments, that they arise from the animal or vegetable kingdom.

The *albumen* and *fibrine* of the blood come from the chyle and lymph. It is observed that the quantity of albumen increases in the chyle during abstinence, whilst that of the fibrine diminishes in the blood and in the lymph.

The *hematosine* is formed probably during the circulation and independently of the spleen, since the removal of this organ produces no change in the colour of the blood. With respect to the manner in which the colouring matter is formed but little satisfactory is known. The same may be said of the other matters contained in the blood. The carbonic acid gas is formed by the combination of oxygen with the carbon of the blood in the capillary vessels.

*Physiological Results.*—The blood yields all the materials of *nutrition*, and the part of the blood which yields them is the liquor sanguinis. Of these materials the *fibrine* occupies the most important place; the blood is no longer coagulable in abstinence. The muscular fibre is almost entirely composed of fibrine. Defibrination brings death with it. *Albumen* and the *fats* chiefly enter into the combination of the nervous system: albumen is probably transformed into fibrine. The *liquid fats* are deposited in the adipose system.

Bichat considered venous blood unfit for the secretions. It must be observed, however, that several secretions, as those of the lungs, liver, (and in some vertebrate animals with cold blood,) those of the kidneys, all come from venous blood. Fat is deposited in greater quantity in men whose blood is less oxidized, whilst nutrition and secretion do not appear at all disturbed. Animal heat appears to be intimately connected with the oxidation of the blood. All the vitality of the nervous and muscular systems seems to depend on the contact of the blood. This is proved by the effects of tying the blood-vessels, and also of diminishing the total mass of blood. When the blood is arrested in any part, this part loses its sensibility: the muscles are no longer subject to the will; they lose their characteristic property of irritability. When the brain is deprived of blood, death follows immediately. Venous and lymphatic absorption cease the moment the arteries are tied. All nutrition, all secretion becomes abolished. Gangrene occurs in parts wholly deprived of blood. Poisons no longer exercise their deleterious effects. The temperature of the part falls. Dropsical effusion takes place. With respect to the effects of excessive abstractions of blood, they are syncope, convulsions, delirium, coma, general debility, dropsy. Repeated blood-lettings produce in the composition of the blood changes which are important in a therapeutic point of view. These changes probably produce others in the density of the blood-vessels, so that the normal state of endosmosis and exosmosis becomes changed. The blood we know furnishes also the materials of secretion. Another question is, whether the liquids secreted exist ready prepared in the blood, or whether the latter undergoes changes in the glands? MM. Dumas, Prevost, Chevreul, &c., are of the former opinion. It is known that the blood contains colouring matters, fats, urea, cholesterine, &c. Urea has been found in the blood of animals, from which the kidneys have been removed, and also in that of the human subject after a retention of urine, for instance in cholera. Without then expressing a judgment against the changes which these chemical elements may still undergo, and probably do undergo, in these glands, and against the particular attraction which presides in each gland over the choice of the liquid secreted, it may be affirmed that the matters secreted exist ready prepared in the blood.

Setting out from this principle, and proving that the lungs should be set down as glands, and resting our argument on the presence of gas in the blood, the author endeavours to prove (*Arch. Gen. de Medecine,*

*Feb.* 1842) that the respiration should be considered as a secretion of the gas dissolved in the blood. Some new researches of Liebig, however, in opposition to those of Magnus, tend to prove that the oxygen is not dissolved in the blood, and that the carbonic acid gas obtained by Magnus is but the product of the change of the bicarbonate of soda into carbonate by hydrogen. The secretion of the carbonic acid gas would then occur only at the very moment of the passage of the blood through the lungs. The expired air contains less oxygen than the inspired air, but more carbonic acid gas. Still the increase of the latter is not in proportion to the quantity of oxygen lost, since more oxygen is absorbed in respiration than is necessary for the formation of the carbonic acid gas expired. With respect to the *pathological results* obtained from the chemical and other examinations of the blood, the author admits that very little has been as yet accomplished. The buffy coat in particular presents itself under such various and even opposite states of the system, that little useful information in the treatment of disease can be obtained from its inspection.

The phenomena of *congestion* and *inflammation* find an explanation in what we know concerning the blood. In these morbid states, says the author, we first see a diminution in the diameter of the capillary vessels, or acceleration of the current. The capillaries then dilate; the blood circulates more slowly, but in a uniform manner. At a later period, the circulation becomes irregular, the blood advances and recedes, it oscillates, and at length is completely arrested. The vessels give way and extravasations of blood take place. At the same time that the blood is arrested, there is transudation, first, of bloody serum, then of the liquor sanguinis, into the surrounding tissue—these phenomena, with some others, form the chief signs of inflammation: redness, pain, swelling. From the moment the blood is arrested commences the second stage of inflammation, called *stasis*. It is at this stage that the extravasation of entire blood is observed, that is, we see the blood-globules in the sputa of pneumonia, in the urine, &c. Now these globules not being capable of passing through the parietes of the capillaries, the latter are supposed to be torn. This stage is soon followed by the third, *exudation*. If it is only bloody serum that transudes, there follows an accumulation of albuminous liquid, containing however less albumen than serum. If, on the contrary, the liquor sanguinis passes through the capillaries, the transuded liquids will contain fibrine. This generally coagulates and thus forms what is called hepatisation, false membranes, &c. Inflammation may terminate by resolution, gangrene and suppuration.

The extensive notice which we have given of this work, whilst it sufficiently indicates the high opinion which we entertain of it, prevents us from continuing our analysis any further. Its happy combination of theory and practice places it far above any work on the same subject with which we are acquainted. All the best modern works we find have been consulted and turned to the best account in its construction. We recommend it therefore to the perusal of those who wish to make themselves acquainted with the important improvements recently made in General Anatomy, more especially in its application to Pathology.

